# A Review of Non-Destructive Raman Spectroscopy and Chemometric Techniques in the Analysis of Cultural Heritage

**DOI:** 10.3390/molecules29225324

**Published:** 2024-11-12

**Authors:** Burak Yogurtcu, Nur Cebi, Anıl Tevfik Koçer, Azime Erarslan

**Affiliations:** 1 Department of Conservation and Restoration of Artworks, Faculty of Fine Arts, Mimar Sinan Fine Arts University, 34427 Istanbul, Turkey; 2 Food Engineering Department, Chemical-Metallurgical Faculty, Yıldız Technical University, 34210 Istanbul, Turkey; 3 Health Biotechnology Joint Research and Application Center of Excellence, 34210 Istanbul, Turkey; tevfik.kocer@yildiz.edu.tr; 4 Bioengineering Department, Chemical-Metallurgical Faculty, Yıldız Technical University, 34210 Istanbul, Turkey; azime@yildiz.edu.tr

**Keywords:** Raman, cultural heritage, chemometrics, pattern recognition, micro-Raman, FT-Raman

## Abstract

Today, there is an increasing concern and effort for protection, conservation, and restoration of cultural heritage materials. Non-invasive analytical methodologies such as Raman spectroscopy offers various advantages such as high speed, robust identification, low cost, and in-site analysis. Previous contributions highlighted the potential of Raman spectroscopy combined with multivariate statistics for identification and quality evaluation of cultural heritage materials such as paints, fiber, dyes, woods, stones, inks, and textile materials. Especially, application of chemometrics and multivariate statistics algorithms opens new horizons for scientists and inspectors. In conclusion, the paper provided an overview of the state-of-the-art uses of multivariate statistically equipped Raman spectroscopy methods for evaluation of cultural heritage and art materials with illustrations from previous research studies.

## 1. Introduction

Today, there is an increasing concern about the scientific evaluation and protection of cultural heritage materials by collecting required information about their properties to hand down cultural heritage to the public [1]. Analytical chemistry comprises a wide variety of instrumental analysis techniques that can be used as effective tools for protection of cultural heritage in the process of restoration (early detection, restoration, evaluation of degradation, etc.) [2]. The primary concepts essential for the protection of cultural heritage materials include determining the impact of environmental factors, identifying chemical reactions occurring in surrounding environments, advancing new treatment processes, and disseminating knowledge among stakeholders [3]. The application of robust analytical techniques, including in situ and complementary methodologies, can provide detailed and valuable information essential for addressing cultural heritage conservation challenges [4].

However, there is a growing recognition of the need for non-destructive methods that can provide detailed information without causing damage to these often fragile materials. This has led to the increasing use of Raman spectroscopy in cultural heritage studies, due to its ability to analyze materials in situ and without requiring sample removal [1]. Raman spectroscopy has unique advantages over other spectroscopic techniques, such as infrared spectroscopy or X-ray fluorescence, as it can provide molecular information and detailed chemical compositions of pigments, binders, and degradation products [3]. Furthermore, it is non-invasive, allowing repeated analysis of the same sample over time, which is crucial for long-term conservation strategies. Additionally, unlike infrared spectroscopy, Raman is less susceptible to interference from water, making it particularly useful in humid environments where heritage objects may be stored or exhibited [5].

The integration of chemometrics further enhances the potential of Raman spectroscopy by enabling the analysis of complex datasets and revealing hidden patterns that may not be immediately apparent in raw spectral data. Together, these methods allow for the discrimination of materials, identification of degradation patterns, and even the mapping of different layers in stratified materials, such as painted surfaces or manuscripts [6].

The protection of cultural heritage materials requires a multidisciplinary approach to develop comprehensive solutions. In this respect, analytical chemistry can be considered as a powerful discipline which is capable of evaluation of chemical composition, physical properties, unique fingerprint properties, and environmental fluctuations [1]. The diagnosis of the materials is quite important to obtain dependable details on the structure of the materials, and this information can be further used for preservation and protection through the enhancement of the lifespan of masterpieces. Various analytical techniques are employed to characterize cultural heritage materials depending on the specific problem at hand. These techniques include atomic and molecular spectroscopic methods such as FTIR, Raman, and reflectance spectroscopy, as well as chromatographic, thermal, and other miscellaneous techniques [5].

Raman spectroscopy, in particular, has gained prominence in cultural heritage preservation due to its versatility and precision. Its ability to operate with minimal or no sample preparation, combined with its high spatial resolution, makes it ideal for delicate heritage materials where invasive techniques are not feasible [4]. Moreover, Raman spectroscopy can provide information at a microscopic level, offering insights into the molecular structure of materials, even in multi-layered and heterogeneous samples. The non-invasive nature of the technique allows for ongoing monitoring of conservation efforts without risking damage to the artifacts [1].

Chemometrics is an interdisciplinary field that combines mathematical, statistical, and computational sciences to optimize the analysis and interpretation of chemical data [6]. It plays a crucial role in processing and making sense of large and complex datasets obtained from techniques such as Raman spectroscopy [7]. In Raman spectroscopy, chemometric methods are particularly valuable for analyzing complex mixtures or when spectral bands overlap, simplifying the data analysis process. Key chemometric techniques include principal component analysis (PCA), partial least squares regression (PLS), linear discriminant analysis (LDA), and hierarchical cluster analysis (HCA) [8]. These methods reduce the dimensionality of large datasets, allowing researchers to identify key differences and correlations between samples. Chemometrics aids in more accurate determination of molecular structures and in quantifying the amounts of compounds based on the information extracted from Raman spectra [9]. PCA, for instance, identifies the main variations in spectral datasets by breaking them down into principal components, while PLS models the relationships between chemical properties and spectral data, improving the accuracy of quantitative analysis [10]. Techniques like LDA are effective in classification and identification processes, helping to distinguish the spectral fingerprints of different samples [11]. Additionally, advanced chemometric methods such as multivariate curve resolution (MCR) allow for the deconvolution of complex spectra, facilitating the separation of different components in mixtures [12].

In particular, the combination of Raman spectroscopy with chemometric techniques enhances the ability to analyze and interpret complex datasets, making it easier to identify degradation processes and original material compositions in cultural heritage objects. Chemometrics provides the ability to handle overlapping spectral features and offers robust statistical methods for classifying and differentiating materials based on their molecular structures. This is essential for analyzing pigments, binders, and varnishes in historical artifacts, where materials often degrade or change over time [6].

In this review, we will focus on the application of Raman spectroscopy as a non-destructive, non-invasive molecular technique that, when combined with chemometrics, holds great potential for the protection of cultural heritage materials. Raman spectroscopy is particularly advantageous due to its ability to perform rapid, high-resolution analysis without the need for extensive sample preparation. It can be applied in situ, allowing for on-site investigations, and can also analyze micrometer-sized areas of cultural heritage materials [13]. Furthermore, portable Raman systems make it possible to conduct fieldwork in heritage sites without transporting samples to laboratories, reducing the risk of damage during transport. The integration of chemometric methods enhances the analysis by facilitating the interpretation of complex Raman spectra, improving sensitivity, and enabling more accurate identification of degradation patterns and materials [14]. This combination of Raman spectroscopy with chemometrics provides a powerful toolset for heritage conservation experts, allowing for both the preservation and the detailed understanding of the materials under study.

## 2. Raman Spectroscopy

Indian physicists C. V. Raman and K. S. Krishnan first experienced the Raman scattering effect in 1928. Today, Raman spectroscopy can be described as one of the most powerful rapid spectroscopy techniques due to its rapid, effective, high-throughput, and robust analysis properties [15]. Raman spectroscopy provides fingerprint information on the molecular level with minimal labor and sample preparation [16]. One of the significant advantages of Raman spectroscopy is its ability to analyze samples in aqueous environments without significant interference from water molecules. Unlike other spectroscopic techniques, such as infrared spectroscopy, which are highly sensitive to water content and often experience strong water absorption, Raman spectroscopy is much less affected by water [17]. This is because the Raman scattering cross-section of water is relatively weak, allowing spectral information to be obtained from the target molecules with minimal disturbance from the surrounding water molecules [18]. Spectroscopy science studies the interactions between light and matter. In Raman spectroscopy, the primary focus is on Raman scattering, which is one of the possible interactions of light with matter. Absorbance and transmittance are other types of interactions, but Raman spectroscopy specifically examines the scattering of light that occurs when photons interact with molecules, leading to shifts in the light’s wavelength [19]. When the molecule is excited with the photon the Raman scattering may occur. The scattering with the same frequency is the elastic scattering (the so-called Rayleigh scattering). It is the preferential process. Raman scattering is an inelastic scattering process. It is much weaker. For every inelastically scattered photon there are 10^7^–10^9^ elastically scattered ones. When the frequency (wavelength) of the scattered photon is lower (higher) by the vibrational mode (phonon) frequency, the Stokes process of Raman scattering takes place. In the opposite case, the anti-Stokes process of Raman scattering occurs [5]. The observed Raman peaks are associated with the chemical structure of the molecule and present a specific and characteristic fingerprint of the investigated compounds. These peaks in the Raman spectrum result from the interaction of monochromatic light with the molecule, providing detailed information about its vibrational modes. In addition to its molecular analysis capabilities, Raman spectroscopy is also one of the most essential technologies for evaluating cultural heritage materials directly in the field. On-site Raman spectroscopy can be applied to various cultural heritage materials such as rock art, paintings, glass objects, porcelains, ceramics, mosaics, manuscripts, sculptures, and altars [13].

### 2.1. FT-Raman Spectroscopy

As a rapid, non-destructive, and high-throughput vibrational spectroscopy technique, Raman spectroscopy reveals the unique fingerprint properties of investigated compounds [16]. Non-destructive analysis does not create any destruction or damage during the experimental procedures. FT-Raman spectroscopy is designed to gather measurements that are stable in terms of wavelength, meaning that the emitted Raman signal remains consistent and reproducible over time, which is crucial for accurate analysis [18]. While FT-Raman spectroscopy significantly reduces fluorescence interference by using a 1064 nm Nd:YAG laser (neodymium-doped yttrium aluminum garnet), it does not entirely eliminate fluorescence. This laser wavelength, however, is highly effective in minimizing fluorescence, especially compared to shorter wavelengths, making it suitable for a wide range of samples. A typical FT-Raman spectrometer consists of an excitation source (Nd:YAG laser), a sampling compartment, an interferometer with fixed and moving mirrors, and a detector [20]. The main component of an FT-Raman spectrometer is known as the Michelson interferometer. The general structure of a Michelson interferometer is constructed by a fixed and moving mirror and a beam splitter. The detector captures the interference pattern generated by the combination of the transmitted light from the fixed mirror and the reflected light from the moving mirror, then converts this pattern into an electrical signal that can be visualized on a computer screen [21]. Chemical specificity, rapid analysis, simple sample preparation, cost-efficient operation, and non-invasive sample handling make FT-Raman spectroscopy a highly effective tool for quality analysis across a wide range of materials, including food and pharmaceuticals [22].

### 2.2. Raman Imaging

Vibrational spectroscopy studies the interaction between light and matter molecules. Observed vibrational bands at certain wavenumbers are specific to the excited molecules, as these bands are formed based on the chemical structure of the compounds. It is possible to mention that the obtained spectrum includes unique spectral information of the molecules at the related wavenumbers [23].

Raman chemical imaging technology integrates the advantages of Raman spectroscopy and digital imaging technology for the evaluation of the chemical composition of materials [24]. This technique allows for spatially resolved chemical information to be obtained, meaning that it can provide detailed images of the distribution of chemical components across a sample. By combining the molecular specificity of Raman spectroscopy with the visual capabilities of imaging, it enables the identification and localization of different substances within heterogeneous samples at microscopic or even nanoscale levels, making it particularly valuable for complex materials analysis [25]. Another advantage is that the Raman spectroscopy provides visualization of the compositional properties of the heterogeneous matrices with scanning properties at micro- or nano-scale [26]. The compositions of cultural heritage materials are complex and can consist of resin, pigment, plasticizer, wood, mosaic, bricks, oils, fibers, and dyes [27].

The spatial resolution in Raman mapping is a critical aspect of the technique, as it defines the smallest detectable chemical feature within a sample [28]. High spatial resolution enables the differentiation of materials that are located very close to one another or even within the same micro-region. In Raman imaging, spatial resolution is typically determined by the diffraction limit of the laser, which is dependent on the wavelength of the laser and the numerical aperture of the objective lens [29]. This means that the spatial resolution typically ranges from the micrometer scale down to the sub-micrometer scale. For example, in applications such as the analysis of cultural heritage artifacts, subtle differences in the composition of pigments or surface coatings can be detected, helping conservators identify and map degradation products or restoration materials without damaging the artifact [30]. Similarly, in biological samples or complex material systems, Raman mapping can reveal the distribution of different molecular species within cellular structures or composite materials, offering insights into chemical heterogeneity that would otherwise be difficult to observe [31]. The spatial distribution of the compounds in a selected sample region can be displayed using the Raman mapping concept. Raman mapping scans across a grid of points on the X and Y axes, gathering Raman spectra at each coordinate [32]. The system, equipped with a conventional motorized stage, allows for high-throughput analysis of micro-samples and cross-sections of materials. This scanning capability, combined with high spatial resolution, allows researchers to create detailed chemical maps that show how the composition varies across a sample [19]. Achieving high spatial resolution is essential for uncovering fine structural details and chemical gradients within a sample. In fields like forensic science, food chemistry, and medical research, this level of detail can provide critical information for quality control, material identification, and diagnosis. For instance, in forensic applications, high-resolution Raman mapping can differentiate between trace substances or contaminants found at a crime scene. In food chemistry, it can be used to verify the homogeneity of a product or detect adulterants at very low concentrations. In medical research, it provides insights into the molecular composition of tissues or cells at a near-cellular level, enabling early detection of diseases [33].

### 2.3. SERS Spectroscopy

In general, one of the most well-known drawbacks of Raman spectroscopy is a weak Raman signal that could not be useful for determining low amounts of analyte. However, in some situations, there is a need for detection and quantification of low amounts of compounds producing low Raman signal. Surface-enhanced Raman spectroscopy (SERS) has high potential to combine fingerprint specificity and single-molecule sensitivity; increased sensitivity provides the opportunity for detection of low or trace amounts of molecules in the chemical and biochemical analysis [34]. SERS technology utilizes metallic nanostructures (SERS substrates: noble metal nanoparticles, rough metal surfaces) to enhance the low-quantity related Raman signal [35]. SERS is based on the enhancement of Raman scattering that occurs when molecules are adsorbed onto a nanostructured noble-metal surface, such as gold (Au) or silver (Ag). This enhancement is primarily due to the excitation of localized surface plasmon resonances (LSPRs) on the metal surface when exposed to a specific wavelength of laser light [36]. The localized surface plasmons are collective oscillations of the metal’s conduction electrons, which create intense electromagnetic fields at the surface. These enhanced fields significantly amplify the Raman signal of molecules in close proximity to the metal surface. For Au and Ag nanoparticles, this plasmon resonance typically occurs in the visible region of the spectrum, leading to strong signal enhancement [37]. Previous studies reported that cultural heritage studies have included the usage of SERS spectroscopy on the colorants (natural or synthetic), natural resins, and daguerreotypes [38,39]. The advantages of SERS include molecular specificity, increased sensitivity, lower detection limits, and the ability to achieve high spatial resolution, meaning that it can provide detailed chemical information from very small or localized areas of a sample, down to the nanometer scale [38].

In addition to surface-enhanced Raman spectroscopy (SERS), there is also surface-enhanced resonance Raman spectroscopy (SERRS), which is particularly effective for the study of colorants and dyes [40]. SERRS occurs when the molecule of interest absorbs light at or near the excitation wavelength, resulting in resonance Raman enhancement. When this resonance condition is combined with the surface plasmon resonance from the metal nanoparticles (as in SERS), the Raman signal is further amplified, often by several orders of magnitude. This dual enhancement makes SERRS a highly sensitive technique for detecting even trace amounts of colorants or pigments in complex mixtures [41].

SERRS is particularly valuable in fields like cultural heritage conservation, where the detection and analysis of pigments and dyes are critical. For instance, in the analysis of historical artifacts, SERRS can be used to identify the composition of pigments with exceptional sensitivity, even in cases where the sample size is extremely small or the pigment is heavily degraded [42].

### 2.4. Raman Spectroscopy with Pulsed Laser Excitation

Raman spectroscopy can also be performed using pulsed laser excitation in addition to the more commonly used continuous wave lasers. Pulsed lasers emit short bursts of high-energy light, offering several advantages in specific applications. This approach becomes particularly effective when combined with laser-induced breakdown spectroscopy (LIBS), as it allows for simultaneous Raman spectroscopy and LIBS measurements within the same device [43]. The use of pulsed lasers enables higher peak power, which enhances the intensity of the Raman signal. Compared to continuous wave lasers, the short bursts of light from pulsed lasers allow for higher energy densities, resulting in stronger Raman signals. Additionally, since the laser is active for only very short durations, the risk of heating or damaging the sample is significantly reduced. This feature is particularly advantageous when working with sensitive samples [44].

When combined with LIBS, pulsed lasers provide the potential for a more comprehensive analysis. LIBS offers elemental composition information by generating plasma, while Raman spectroscopy provides molecular-level insights [45]. This combination allows for a holistic understanding of both the elemental and molecular components of the sample. The combined use of these techniques is especially valuable in cultural heritage studies, where the analysis of complex materials such as pigments and coatings is required. This approach enhances the ability to study such materials with greater depth and accuracy, offering a critical tool for both scientific research and preservation efforts [43,45].

## 3. Raman Spectroscopy Applications on Cultural Heritage Materials

Today, Raman spectroscopy can be mentioned as a reliable analytical methodology for the chemical characterization and evaluation of cultural heritage materials. The superiorities of Raman spectroscopy in terms of high spatial resolution, molecular specificity, non-invasive analysis, imaging and mapping opportunities, and rapid analysis make the technique an effective alternative for cultural heritage materials [13]. Raman spectroscopy can be applied effectively for the investigation of a broad range of materials, including organic and inorganic pigments, varnishes, plastics, glass, and ceramics. The inspection superiorities make Raman spectroscopy a multi-functional and essential tool in cultural heritage materials [46]. The application of Raman spectroscopy combined with chemometrics assists scientists by opening new horizons in conserving cultural heritage materials. Especially, on-site and in-the-field applications make Raman spectroscopy capable for rapid and cost-effective evaluation of quality properties of cultural heritage materials [47]. Practical Raman applications in the cultural heritage field could be categorized under these headlines according to the material types: (a) pigments, inks and colorants, (b) minerals and gemstones, (c) natural and synthetic organic materials, (d) deterioration products and conservation treatments, (e) glass, and (f) ceramics [37]. This study aimed to explain the potential of Raman spectroscopy combined with chemometrics for investigating cultural heritage materials. This review article mainly focused on the application of Raman spectroscopy techniques equipped with multivariate statistics in the field of cultural heritage materials. In this context, Table 1 presents the related previous contributions for the quality evaluation of various cultural heritage materials, from painting to oil types.

These studies included the applications of Raman spectroscopy, FT-Raman spectroscopy, Raman–LIBS, and Raman imaging system in combination with chemometrics. Most of these studies investigated the painting materials and dyes. Significantly, the chemical compositions of the painting materials are complex, requiring elaborate characterization approaches such as the GC–MS technique. Manzano et al. [48] explored the use of Raman spectroscopy combined with chemometric methods for the discrimination of aged mixtures of lipidic paint binders. The study investigated common lipid binders such as linseed oil, poppy seed oil, walnut oil, and egg yolk. The experiments were conducted using a Bruker RFS FT-Raman spectrometer with a 1064 nm Nd laser at a power of 100 mW. Spectra were recorded with a resolution of 4 cm^−1^ over 250 scans. The comparison between fresh and aged samples showed significant spectral changes, particularly at 1267, 1655, and 3011 cm^−1^, which correspond to the vibrations of cis double bonds. These changes were indicative of the aging process. The model samples used in the study consisted of pure binders and binary mixtures of these binders, which were naturally aged for 6 years at room temperature. Multivariate analysis methods such as principal component analysis (PCA) and partial least squares discriminant analysis (PLS-DA) were applied to the Raman spectra to determine whether spectral differences could differentiate the samples based on their compositions. The results demonstrated that multivariate methods like PCA and PLS-DA were successful in distinguishing the aged samples based on their lipid binder compositions. Particularly, the C–H stretching vibrations at 2893 and 2854 cm^−1^ were identified as regions with high discriminative power [48]. Analytical methodologies are needed to be applied for protection of cultural heritage in the different stages of the restoration process (evaluation of degradation, early detection, restoration, and monitoring). Romani et al. [3] applied Raman spectroscopy and chemometrics to the study of Vincenzo Pasqualoni’s wall paintings in the apse of S. Nicola in Carcere, Rome. The Raman analysis was performed using a B&W TEK Inc. (Newark, DE, United States) Raman spectrometer equipped with a 785 nm GaAlAs diode laser. The laser power was adjustable between 3 to 300 mW, and a 2048-pixel CCD detector cooled to 10 °C was used for signal detection. The spectral range covered was 75 to 3200 cm^−1^, with a spectral resolution of approximately 3.5 cm^−1^. In situ measurements were performed using a fiber-optic Raman probe, with the laser beam focused to a 90 μm spot size at a working distance of 5 mm. Exposure times ranged from 9 to 18 s per spectrum to prevent photodecomposition of pigments, and spectra were averaged over three scans to enhance the signal-to-noise ratio. Using chemometric approaches such as PCA and PLS-DA, the authors successfully identified the original pigment palette and documented the use of various binders and protective materials. The study revealed that the artist predominantly used ochers, chrome green, and cobalt blue. The PLS-DA model achieved high sensitivity and specificity in pigment classification. However, limitations such as the potential degradation of pigments and the influence of restoration materials were noted. The combination of Raman spectroscopy with chemometric techniques demonstrated its high potential for non-invasive and accurate pigment identification in cultural heritage studies. The study also showed the importance of complementary techniques like X-ray fluorescence (XRF) and infrared spectroscopy to corroborate the findings and address limitations such as signal interference from overlapping pigment layers [3]. Clearly, classification of pigments and extraction of characteristic spectral information through multivariate statistics is a popular application due to their effectiveness on these challenging problems. Coherently with this topic, Festa et al. [53] applied Raman spectroscopy and chemometrics to a set of 48 powdered pigments and investigated a real case study from a wall painting in S. Maria Delle Palate di Tusa, Messina, Italy. For Raman measurements, a BRAVO Raman spectrometer was used, equipped with 785 nm and 853 nm excitation lasers, which enabled the collection of spectra between 300–3200 cm^−1^. The BRAVO system’s fluorescence mitigation capabilities were especially important for minimizing interference, a common issue with certain pigments. Chemometric analysis, primarily PCA, was applied to distinguish between organic and inorganic pigments based on their molecular and elemental benchmarks. The authors employed XRF to measure elemental composition, using an XRaman portable instrument with a detection range of 30–50 keV and a silicon drift detector. Raman and XRF data were combined with PCA to classify pigments, which allowed Festa et al. [53] to create clear groupings for the pigments based on their elemental content, such as the differentiation of Fe-based and Cu-based pigments. The methodology’s strength lies in its ability to analyze a wide range of pigments non-destructively, while PCA proved highly effective in identifying spectral benchmarks that differentiate inorganic and organic pigments. However, limitations were noted, such as the complexity of analyzing pigments mixed with binders and degradation products, which sometimes reduced the clarity of the spectral data. Nevertheless, this study successfully demonstrated the utility of combining Raman spectroscopy with chemometrics for cultural heritage preservation [53].

In another study, Navas et al. [54] explored the capabilities of Raman spectroscopy combined with PCA for evaluating historical tempera paint model samples. The study used a Renishaw Invia Raman microscope system with a 514.5 nm argon ion laser (Laser Physics, model 23S514), and a Peltier-cooled CCD detector. The laser power was kept between 0.2 to 20 mW to prevent degradation of the sensitive pigments, and spectra were collected using a 20× and 50× long-working-distance objective. Raman spectra were recorded in the range of 200–3800 cm^−1^, with an exposure time of 20 s per scan, and the scans were averaged to improve signal-to-noise ratios. Spectra were pre-treated using first-derivative transformation, which significantly improved the ability of PCA to discriminate between samples based on their pigment composition. The pigments used for the model samples included blue pigments like azurite, lapis lazuli, and smalt, red pigments like cinnabar, minium, and raw sienna, and white pigments like lead white, chalk, and gypsum, mixed with egg yolk as a binder. PCA was applied to Raman spectra to test whether these samples could be distinguished by their spectral features. The results showed excellent discrimination between the samples based on the pigments’ chemical compositions. Specifically, PCA was highly effective in identifying the lead white and gypsum samples based on the spectral bands in the range of 1200–600 cm^−1^ for white pigments, and in the range of 1100–600 cm^−1^ for blue pigments. While the study successfully demonstrated the potential of Raman spectroscopy and PCA for differentiating complex paint compositions, limitations were noted in detecting degradation of pigments like minium, which showed signs of breakdown under laser exposure. Despite this, the study highlights Raman spectroscopy’s strength as a non-destructive analytical tool in the field of cultural heritage, especially when combined with multivariate chemometric methods like PCA [54].

In a different study, Zou et al. [64] evaluated the potential of portable Raman spectroscopy for the identification of different kinds of watercolor inks in forensic science. The study used an InnoRam portable research Raman spectrometer with a laser wavelength of 785 nm, operating at a laser power of 300 mW, and covering the spectral range of 65–3250 cm^−1^. The spectrometer provided a spectral resolution of 2.0 cm^−1^. To ensure reliable results, each watercolor ink sample was measured three times, and the average of the spectra was taken for further analysis. The ambient temperature for all measurements was maintained between 25–27 °C to ensure stability. Spectral data preprocessing included multiple scattering corrections and Savitzky–Golay smoothing (five points), which were applied to reduce noise and improve the quality of the acquired spectra. The study applied mid-level data fusion, combining Raman and FTIR spectroscopy data, along with chemometric techniques, such as PCA and PLS-DA, to improve classification accuracy. The results demonstrated that mid-level data fusion effectively complemented the strengths of both Raman and FTIR spectroscopy. Specifically, the classification model using the VCPA-IRIV algorithm achieved a 100% classification accuracy in identifying different watercolor ink brands. This fusion approach proved superior to using either Raman or FTIR data alone, highlighting the method’s ability to provide comprehensive chemical information about both pigments and binders. One limitation noted was that while the mid-level data fusion model performed well, noise and uninformative variables present in the raw data could reduce the overall accuracy if not properly filtered. The VCPA-IRIV algorithm was instrumental in overcoming this limitation by selecting the most relevant variables from the dataset [64]. Another essential concept in the analytical chemistry field is the determination of fragrance content in the perfumes for classification of them according to the guidelines established by the perfume manufacturers in a rapid, non-destructive, and sustainable manner. Godinho et al. [68] developed an alternative methodology for the determination of fragrance content in perfumes and their classification using Raman spectroscopy and multivariate calibration. The study used a PerkinElmer RamanStation 400F spectrometer with a 785 nm laser excitation source operating at 250 mW. The spectrometer was equipped with a two-dimensional charge-coupled device (CCD) detector, which was cooled to −50 °C to reduce thermal noise during acquisition. Samples were placed in 2 mL closed glass vials without any pre-treatment and analyzed over a spectral range of 200–3278 cm^−1^, with four scans per sample and a spectral resolution of 2 cm^−1^. The spectra were processed in real time using PerkinElmer Spectrum 6.3.5 software. Multivariate calibration methods, including PCA and PLS, were employed to correlate the Raman spectra with the fragrance content of the samples. The model was validated using both leave-one-out cross-validation and external validation sets. The results demonstrated that Raman spectroscopy, combined with multivariate data processing, provided a reliable prediction of fragrance content in commercial products, comparable to results obtained using GC–MS. The standard error of prediction (SEP) was 0.47%, which highlights the method’s accuracy for commercial perfume analysis. Moreover, PCA allowed for the identification of ethanol content in the fragrance samples, distinguishing between different fragrance concentrations and ethanol–water ratios. While the study demonstrated the advantages of using Raman spectroscopy for its rapid, non-destructive, and solvent-free analysis, a limitation noted was the overlapping of C–H stretching bands from different fragrance molecules, particularly in the region around 1450 cm^−1^, which could affect the clarity of certain spectral features. Despite this, the methodology proved to be a valuable tool for quality control and regulatory purposes in the perfume industry [68].

A different contribution proposed a new in situ Raman methodology for quantification of polyethylene glycol (PEG) in waterlogged wood and calibration models were developed using OPLS (orthogonal partial least squares); their results showed that quantification of PEG content was successfully accomplished for archeological wood and raw wood samples between the spectral range of 3200–200 cm^−1^. The authors stated that the method is reliable on the basis of the developed validation model [71].

Rock art pictographs are important cultural heritage materials and the identification of used pigments is important for protecting and restoring these arts. Pitarch et al. [72] demonstrated the combined use of Raman spectroscopy and energy dispersive X-ray fluorescence (EDXRF) as an effective method for the in situ analysis of post-Paleolithic black pictographs in the Los Chaparros rock shelter, a UNESCO World Heritage Site. The study used a portable InnoRam Raman spectrometer equipped with a 785 nm diode laser and a hand-held X-MET5100 EDXRF analyzer, B&W TEK INC., Newark, DE, USA. The Raman system covered a spectral range from 65 to 2500 cm^−1^, with the laser power not exceeding 9.5 mW at the focus point to prevent any alteration to the analyzed areas. Spectral acquisition was carried out with an integration time varying between 0.5 to 10 s, and the number of accumulations ranged between 20 to 200, depending on the presence of fluorescence and the signal-to-noise ratio. The EDXRF analyzer was used to identify the elemental composition of the pigments through non-destructive, in situ measurements, with a spectral range from 0 to 40 keV. The black Levantine deer and red schematic pictographs were analyzed, and PCA was applied to EDXRF spectra to investigate the elemental differences between manganese dendrites, the black pigment, and the rock substrate. The Raman analysis revealed the presence of Mn–O and Mn–OH bending and stretching vibrations, confirming the use of manganese-based black pigments. Laboratory analyses using micro-Raman spectroscopy and XRD on mineral samples confirmed the in situ findings, showing a strong similarity between the manganese dendrite mineralizations found at the site and the pigments used in the pictographs. One limitation of the study was the low Raman signal from manganese compounds, which made it difficult to obtain clear spectra in some areas. However, the combination of EDXRF and Raman spectroscopy provided complementary data that were essential for the identification of both elemental and molecular components of the pictographs. The study emphasized the utility of non-invasive, portable spectroscopic techniques for fieldwork in heritage conservation, allowing detailed analysis without damaging the artifacts [72].

In cultural heritage materials, pigment identification is extremely crucial for the conservation, protection, and preservation of the art materials. The thesis study of Gonzalez Vidal [74] evaluated the capability of different chemometrics strategies to eliminate complexities arising from the interferences of mixed pigments for accurate pigment identification. Their results showed that developed methodologies had capabilities for the identification of pigments correctly in the pigment mixtures by using fingerprint Raman spectral properties. Additionally, the authors noted the robustness of the technique against critical factors which may have an impact on pigment identification by the Raman methodology [74].

Peris-Diaz et al. [49] implemented FT-Raman spectroscopy coupled with PLS to discriminate the provenance and geological age of amber samples. The study used a Nicolet Magna 860 FT-IR spectrometer (Waltham, MA USA) with a 1064 nm InGaAs laser for Raman excitation. Spectral data were collected at 23 °C over a range of 100 to 3800 cm^−1^ with a spectral resolution of 4 cm^−1^ and a scan rate of 512 measurements per sample. Laser power was set below 0.8 W for each sample to minimize thermal effects. Amber samples, including succinite and valchovite, were analyzed without preliminary preparation, and Raman spectra were collected in triplicate from multiple points on each sample to provide averaged data. To model the provenance of the amber, the spectral data were processed using multiplicative scatter correction (MSC) followed by auto-scaling. For geological age modeling, the spectra were subjected to asymmetric least squares baseline removal and then scaled by mean centering. Chemometric analysis through PLS and PLS-DA demonstrated a strong ability to discriminate between Baltic (succinite) and Czech (valchovite) amber, with a Q^2^Y value of 0.915 for the full spectral range and 0.892 for the 1100–1800 cm^−1^ interval. Additionally, the ratio of the I1645/I1450 cm^−1^ bands, often used to assess the maturity of fossil resins, proved reliable in this study for differentiating samples by age. The study identified significant wavenumbers for discrimination, with bands at 972 cm^−1^ (CH_2_ bending), 1450 cm^−1^ (CH_2_ scissoring), and 2800–3000 cm^−1^ (CH_3_ stretching) being particularly important. One limitation noted was the effect of environmental factors, such as weathering layers on the amber surfaces, which could interfere with the spectral data. However, the use of chemometric models like PLS effectively minimized these issues, allowing for robust classification of the amber by both provenance and age [49].

A study by Edwards and Munshi [51] demonstrated the capability of FT-Raman spectroscopy for the non-destructive characterization of various biomaterials, including mummified tissues, resins, wall paintings, frescoes, and ivory. The experiments were conducted using a Bruker IFS 66/FRA 106 Fourier-Transform Raman spectrometer (Billerica, MA, USA) with a 1064 nm Nd laser for excitation. The system had a spectral resolution of 4 cm^−1^, and spectra were collected over the range of 100 to 3500 cm^−1^. The laser power was set to prevent thermal degradation of the samples, particularly sensitive biomaterials like mummified tissues and ancient frescoes. The study highlighted the advantages of FT-Raman spectroscopy, such as its non-destructive nature, the minimal sample preparation required, and the technique’s ability to analyze both organic and inorganic materials simultaneously. The identification of molecular fingerprints through Raman spectroscopy allowed for the tracking of degradation processes in these cultural heritage materials. For example, the study identified characteristic protein and mineral bands in mummified tissue samples, revealing the state of preservation. Resins and wall paintings were also effectively analyzed without damaging the surface layers. One limitation noted was the potential interference from fluorescence, which could sometimes obscure the weaker Raman signals, particularly in the analysis of materials with complex compositions like frescoes. However, by using the 1064 nm laser, the study minimized fluorescence interference, making FT-Raman spectroscopy a reliable tool for analyzing fragile, historical artifacts. The study also suggested that combining Raman spectroscopy with other techniques, such as X-ray fluorescence (XRF) or infrared spectroscopy, could provide complementary data for a more comprehensive analysis [51].

**Figure 1:**
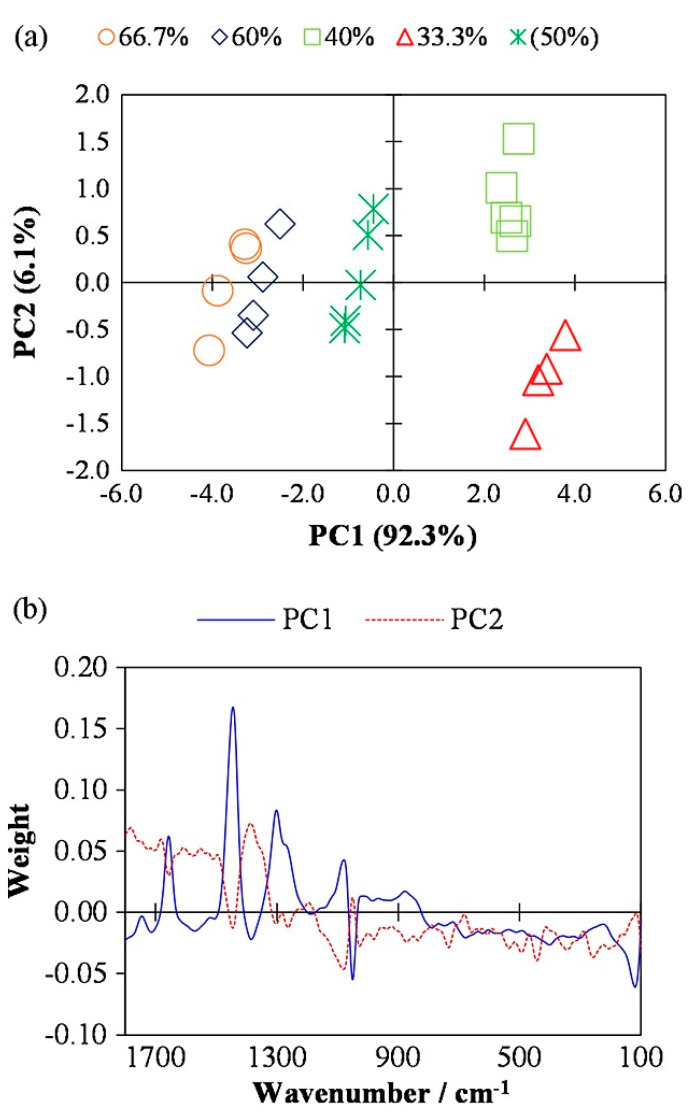
The PCA results of FT-Raman spectra obtained from lead white–egg yolk paint samples (33.3%, 40%, 60%, and 66.7% LW) (**a**). The loading plots of chemometrics of PCA (PC1: 90%, PC2: 6%) (**b**). Reprinted from [56], Copyright (2014), with permission from John Wiley and Sons.

In one of the previous contributions, Pallipurath et al. [56] investigated the potential for prediction of pigment concentration in binders and lead-based dyes using FT-Raman spectroscopy and PCA. The authors stated the importance of the contribution as a first step for a semi-automated and quantitative methodology for quality evaluation of cultural heritage materials to understand their history better and create techniques for their preservation. The results showed that it was possible to use FT-Raman spectroscopy for the estimation of the ratios of pigment/binder in a dye layer [56]. The principle component analysis results of FT-Raman spectra obtained from lead white–egg yolk paint samples are presented in Figure 1a. These sample design sets included 33.3%, 40%, 60%, and 66.7% LW samples. As it can be understood from the PCA graph, the samples including the lowest LW amounts distinctly classified on the third negative region of the PCA graph. Also, the samples including similar amounts of LW % of 60 and 66, which are classified as closely related on the PCA graph. The loading plots of chemometrics are presented in Figure 1b. According to their findings, the first principal component explained over 90% variance and the second principal component explained the 6% of the variance [56].

Pereira et al. [50] assessed the potential of micro-Raman spectroscopy in examining the carbon graphitization process and categorizing ancient inks found in papyri samples spanning several historical eras. They employed a LABRAN HR 800 UV micro-Raman spectrometer (Horiba-Jobin and Yvon Spex, Palaiseau, France) with a 532.1 nm solid-state Nd laser. The spectrometer featured a 1200 lines/mm diffraction grating, covering the spectral range from 150 to 2200 cm^−1^ with a liquid nitrogen-cooled CCD detector. The laser power was adjusted to 270 μW to ensure non-invasive analysis. Each sample underwent spectral collection with integration times ranging from 0.5 to 60 s, and measurements were taken across several spots for each ink sample. Using a five-peak spectral model for deconvolution of the first-order region of the Raman spectra, the researchers investigated the carbon type of soot-based inks. Raman band ratios were evaluated to assess the graphitization process. Multivariate techniques, including PCA and cluster analysis, were employed for ink classification. Fisher’s discriminant functions further improved ink differentiation across historical eras. The study successfully classified the inks by their historical context, confirming the ability of micro-Raman spectroscopy to distinguish between inks from the Roman (1st–2nd century) and Byzantine (7th–8th century) periods. However, some limitations were noted, such as the challenge of analyzing highly degraded samples and environmental factors affecting the Raman signal [50].

**Figure 2:**
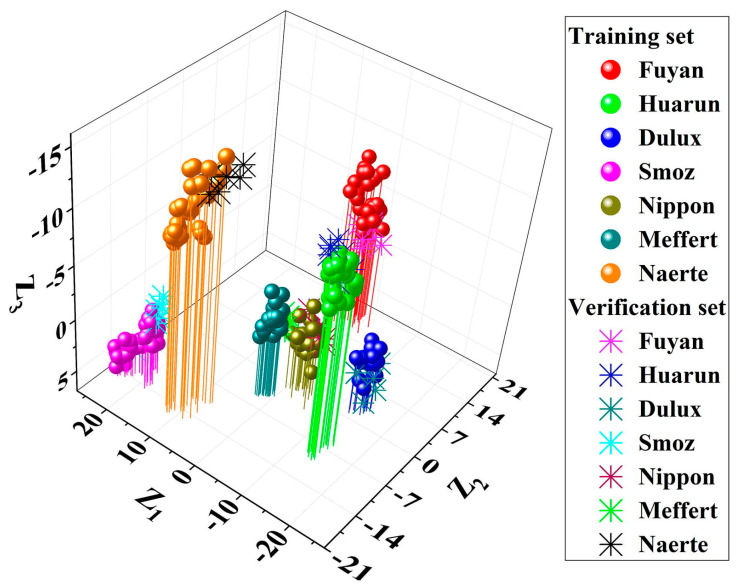
3D discrimination pattern of architectural paints from 7 different manufacturers: Fuyan, Huarun, Dulux, Smoz, Nippon, Meffert, and Naerte. Reprinted from [33], Copyright (2021), with permission from Elsevier.

Another valuable study proposed an effective, strong, cost-effective, and non-invasive microscopic Raman spectroscopy methodology for the discrimination and classification of architectural paints (*n* = 252) from seven different manufacturers. The best pre-processing technique for MLRM data was determined to be the Raman data treated using the fifth Newton interpolation polynomial together with the Savitzky–Golay seven-point and first or second polynomial smoothing under the first-order derivative. According to their findings, all samples were clearly discriminated rapidly and non-destructively. The research paper proved the high potential of micro-Raman spectroscopy combined with multivariate statistics for the development of a reliable and robust methodology for discrimination of white architectural paints which are important for forensic science [33]. A 3D discriminant analysis plot of architectural paints from seven different manufacturers is presented in Figure 2. As can be seen on the plot, clear classification and discrimination between samples were observed on the basis of the manufacturer. Dulux and Smoz samples were clearly discriminated from each other. Littler scattering was observed between Smoz samples, and when compared between each other, the Nippon, Meffert, and Smoz samples were more concentrated.

Wiggins et al. [62] employed Raman imaging and multivariate curve resolution (MCR) methods for polymorph identification in green Chinese architectural paints, particularly focusing on basic copper chloride polymorphs such as atacamite and botallackite. The study utilized a Senterra Raman spectrometer (Bruker Optics, Rosenheim, Germany) with an excitation source of 785 nm. The laser was focused using a 50× objective with a numerical aperture of 0.75, and spectra were collected with a CCD detector cooled to −65 °C. The spectral acquisition covered a range from 80 to 1525 cm^−1^ at a spectral resolution between 3 and 5 cm^−1^. The laser power was 0.1 mW, with an exposure time of 240 s for each measurement. Raman maps were generated by scanning across grids, with step sizes ranging from 3 to 8 microns to produce high-resolution images of the pigment composition. The authors employed multivariate curve resolution–alternating least squares (MCR–ALS) to analyze the Raman spectral datasets, which allowed the resolution of the copper chloride polymorphs. The study revealed that atacamite and botallackite were the primary polymorphs present in the paint samples, and the methodology enabled a detailed visualization of their spatial distribution within the cross-sectioned samples. Additionally, the use of MCR–ALS allowed for accurate separation of weak Raman signals from background noise and fluorescence. One limitation noted was the presence of fluorescence interference, which required additional steps for baseline correction. However, the methodology effectively minimized this interference, allowing for the identification and quantification of the polymorph ratios. The study concluded that this combined approach of Raman imaging and MCR–ALS is a powerful tool for distinguishing and mapping polymorphs in complex materials like architectural paints [62].

In a different contribution, Bianchi et al. [63] employed Raman microscopy and discriminant analysis for the discrimination of aged fibers by Raman spectroscopy and multivariate analysis using PCA and linear discriminant analysis (LDA). The study evaluated cotton, polyester, and polyamide textiles by determining their fiber compositions and specific spectral bands within the 300 to 1700 cm^−1^ range. Raman spectra were collected using a DXR Raman Microscope (Thermo Scientific, Waltham, MA, USA) with two excitation sources, a 532 nm Nd laser and a 780 nm NIR laser diode. For each fiber, the laser power was adjusted to 6 mW for cotton and 15 mW for polyamide/polyester, and data acquisition was performed with a 50× objective using autotiming with an average acquisition time of 120 s per spectrum. The study’s key finding was that discriminant analysis correctly classified aged fibers with 100% accuracy. The leave-one-out cross-validation confirmed that this model could successfully differentiate new, used, and old fibers based on their Raman spectral data. The PCA results explained 64% of the variance in the fiber spectra, while LDA provided a perfect classification for both original and cross-validated samples. The advantage of using Raman microscopy in this context was its non-destructive nature and the ability to analyze fibers mounted on glass slides without requiring sample removal or cleaning, which is often necessary with other techniques such as FTIR. However, fluorescence interference posed a limitation, particularly with polyester and polyamide fibers, which required careful adjustment of laser power and exposure times to minimize this effect [63].

## 4. Conclusions

The national and global importance of cultural heritage materials necessitates the identification of their compositional properties through rapid, reliable, high-throughput, and in situ techniques to support their maintenance, protection, and preservation. Traditional methods for analyzing these materials often involve the use of hazardous chemicals, are time-consuming, and require specialized expertise. However, recent advancements in Raman spectroscopy, including FT-Raman and micro-Raman, combined with sophisticated multivariate statistical methods such as PCA, PLS-DA, and LDA, have demonstrated high potential for overcoming these limitations. In terms of experimental techniques, portable Raman spectrometers have proven particularly effective for in situ analysis, allowing for the rapid identification of pigments, binders, and degradation products directly in the field, without damaging the artifacts. Benchtop Raman spectrometers equipped with 1064 nm Nd lasers have been used to minimize fluorescence interference during the analysis of complex materials, such as organic binders (e.g., linseed oil, poppy oil, and egg yolk) and pigments (e.g., lead white, cinnabar, azurite). Additionally, micro-Raman spectroscopy, with its high spatial resolution, has been employed for detailed mapping of pigment distributions in paint cross-sections and for the characterization of textile fibers (e.g., silk, wool, cotton) in historical textiles.

While Raman spectroscopy and chemometric methods have shown great promise, certain limitations remain. For example, fluorescence interference, though mitigated by techniques such as FT-Raman, still poses challenges in certain materials, particularly those with complex organic compositions. Additionally, the current spatial resolution of portable Raman systems, although improving, remains limited compared to benchtop or micro-Raman instruments. Further research could focus on developing higher-resolution portable Raman systems that combine the advantages of both portability and precision.

Another unresolved issue is the difficulty in analyzing heavily degraded materials, where Raman signals may be weak or obscured by environmental contamination. Future advancements could aim at improving the sensitivity of Raman instruments or developing new algorithms to better interpret noisy or incomplete spectral data. Expanding the range of chemometric methods applied to Raman data, such as incorporating more advanced machine learning techniques, could also offer more robust analysis and classification in complex datasets.

In conclusion, Raman spectroscopy coupled with advanced chemometric techniques has shown groundbreaking potential for the conservation, protection, and restoration of cultural heritage materials. These methods enable the rapid, non-destructive, and reliable characterization of a wide range of materials, including paints, pigments, textiles, resins, and archaeological wood, making them indispensable tools for cultural heritage science. However, addressing the current limitations and continuing to innovate in areas such as fluorescence reduction, resolution enhancement, and the integration of machine learning techniques will be crucial for further advancing the field. These improvements could open up new possibilities for even more detailed and accurate preservation of cultural heritage materials in the future.

## Figures and Tables

**Table 1:** Previous contributions for quality evaluation of various cultural heritage materials using Raman spectroscopy combined with chemometrics.

Detection Technology	Commodity	Samples	Measured Parameters	Spectral Range	Chemometric Methods	Ref.
Raman	Painting materials	Linseed, poppy and walnut oil, egg yolk	Chemical bonds and their change with aging	3800–750	Principal component analysis and partial least squares discriminant analysis	[48]
FT-Raman	Amber	Succinite, valchovite, baltic amber	Intensity ratio of specific bonds for the classification of amber samples according to their provenance and geological age	3800–100	Partial least squares and partial least squares discriminant analysis	[49]
Micro-Raman	Inks	Inks in papyri samples from different historical periods	Ink composition	2200–150	Principal component analysis and discriminant function analysis	[50]
FT-Raman	Biomaterials	Mummified tissue, resins and sourcing of materials, wall-paintings and frescoes, ivories	Specific bands such as carbonate ion, ketonic C=O, alkenic C=C, magnesite, etc., some molecular ions in minerals, pigments, or substrate	3500–100	-	[51]
Microscopic Laser Raman	Paint	White architectural paints	The position, shape, and intensity of spectral peaks	2000–200	Principal component analysis	[33]
Raman	The pictorial-layer materials	Pigments, binders, and protective materials	Specific ions for pigment identification and binder and protective material characterization	3200–75	Principal component analysis and partial least squares discriminant analysis	[3]
Raman-LIBS	Cultural heritage samples	Calcitic and dolomitic marble samples and a patina of calcium oxalate	Nonhydrated compounds in the marble samples		Direct classical least squares	[52]
Raman	Paint	Natural powdered pigments and pigments of the wall painting	Specific bands related to organic and inorganic pigments	3666–2	Principal component analysis	[53]
Raman	Paint	White pigments, blue pigments, red pigments, binder	Characteristic spectral bands of dye pigments	3800–200	Principal component analysis	[54]
FT-Raman	Wood	Wooden boards, pigmented surfaces,	Specific vibrational modes	4000–50	Principal component analysis	[55]
FT-Raman	Paint	Lead white–egg yolk and lead-tin yellow–poppy oil	The intensity values of specific peaks	3600–20	Principal component analysis	[56]
Raman HyperSpectral Imaging	Archaeological samples	Mosaic	Specific shifts for chemical compounds	-	Principal component analysis	[57]
Raman	Wall	Red bricks, yellow bricks	Specific bands related to organic and inorganic pigments	2500–125	-	[58]
Micro-Raman	Documents	Iron-based ink	The intensity values and behaviors of specific peaks	2000–200	Principal component analysis and linear discriminant analysis	[59]
Raman	Historic painting materials	Natural composites and albumin–quartz composites	Molecular, chemical, and mineralogical changes	3200–400	Principal component analysis	[60]
Micro-Raman and FT-Raman	Dyed textile fibers	Silk and wool fibers dyed with turmeric and saffron dyes	Characteristic peaks and degradation of the fiber substrate	3200–100	Principal component analysis	[61]
Raman	Paint	Green and blue architectural pigment	The rough topography of the samples and Raman shifts	1542–80	Multivariate curve resolution–alternating least squares	[62]
Raman	Textiles	Cotton, polyester, and polyamide	Fiber compositions and specific bands	1700–300	principal component analysis, linear discriminant analysis	[63]
Raman	Ink	Watercolor inks	Characteristic shift position and curve shape of spectral peaks	3250–65	Competitive adaptive reweighted sampling, random frog, variable combination population analysis genetic algorithm, and variable combination population analysis–iteratively retains informative variables	[64]
FT-Raman	Wood	Wooden boards	Characteristic components	4000–50	Principal component analysis	[65]
Micro-Raman	Textile fibers	Silk, wool, and cotton	Specific bands	3200–100	Principal component analysis, linear discriminant analysis, and soft independent modeling of class analogy	[66]
Raman Microscope	Painting materials	Azurite, smalt, cinnabar, raw sienna, lead white, chalk, gypsum, and lapis lazuli	Characteristic C–H stretching bands	3400–200	Principal component analysis	[67]
Raman	Perfume	Water–alcohol, standard fragrance, and fragrance samples	Specific bands	3278–200	Principal component analysis and partial least squares	[68]
Raman	Paints	Wall painting materials	Identification of mortar, pigments, and salt composition and distribution	3100–100	-	[69]
Micro-Raman	Dye	Linseed oil paint (lead white and zinc white pigments)	Intensity and changes of specific bands	2000–200	Principal component analysis	[70]
Raman	Wood	Archaeological wood and raw wood	PEG content	3200–200	Principal component analysis and orthogonal projections to latent structures	[71]
Raman	Dye	Black pigments in post-paleolithic blackish pictograph	Specific bands related to blackish pigments	2200–100	Principal component analysis	[72]
Raman	Dye	Carbon-based black pigments	Specific bands related to carbonous structures	2750–60	-	[73]
Raman	Paint	Dye pigments	Specific bands related to different color pigments	2000–200	Principal component analysis	[74]

Shift refers to the Raman shift, which indicates the energy shift resulting from the interaction of laser light with molecules, providing insights into the molecular structure.

## References

[1] Cuca B., Zaina F., Tapete D. (2023). Monitoring of damages to cultural heritage across Europe using remote sensing and earth observation: Assessment of scientific and grey literature. Remote Sens..

[2] Madariaga J.M. (2015). Analytical chemistry in the field of cultural heritage. Anal. Methods.

[3] Romani M., Capobianco G., Pronti L., Colao F., Seccaroni C., Puiu A., Felici A.C., Verona-Rinati G., Cestelli-Guidi M., Tognacci A. (2020). Analytical chemistry approach in cultural heritage: The case of Vincenzo Pasqualoni’s wall paintings in S. Nicola in Carcere (Rome). Microchem. J..

[4] Bertrand L., Schöder S., Joosten I., Webb S.M., Thoury M., Calligaro T., Simon A. (2023). Practical advances towards safer analysis of heritage samples and objects. TrAC Trends Anal. Chem..

[5] Magdy M. (2022). Analytical Techniques for the Preservation of Cultural Heritage: Frontiers in Knowledge and Application. Crit. Rev. Anal. Chem..

[6] Brown S.D. (2017). The chemometrics revolution re-examined. J. Chemom..

[7] Baker M.J., Byrne H.J., Chalmers J., Gardner P., Goodacre R., Henderson A., Sulé-Suso J. (2018). Clinical applications of infrared and Raman spectroscopy: State of play and future challenges. Analyst.

[8] Peris-Díaz M.D., Krężel A. (2021). A guide to good practice in chemometric methods for vibrational spectroscopy, electrochemistry, and hyphenated mass spectrometry. TrAC Trends Anal. Chem..

[9] Spencer P., Ye Q., Kamathewatta N.J., Woolfolk S.K., Bohaty B.S., Misra A., Tamerler C. (2021). Chemometrics-assisted Raman spectroscopy characterization of tunable polymer-peptide hybrids for dental tissue repair. Front. Mater..

[10] Janné K., Pettersen J., Lindberg N.O., Lundstedt T. (2001). Hierarchical principal component analysis (PCA) and projection to latent structure (PLS) technique on spectroscopic data as a data pretreatment for calibration. J. Chemom..

[11] Lasalvia M., Capozzi V., Perna G. (2022). A comparison of PCA-LDA and PLS-DA techniques for classification of vibrational spectra. Appl. Sci..

[12] Ruckebusch C., Blanchet L. (2013). Multivariate curve resolution: A review of advanced and tailored applications and challenges. Anal. Chim. Acta.

[13] Rousaki A., Vandenabeele P. (2021). In situ Raman spectroscopy for cultural heritage studies. J. Raman Spectrosc..

[14] Guo S., Popp J., Bocklitz T. (2021). Chemometric analysis in Raman spectroscopy: From experimental design to machine learning–based modeling. Nat. Protoc..

[15] Liu F., Zou X., Yue N., Zhang W., Zheng W. (2023). Correlative Raman imaging and scanning electron microscopy for advanced functional materials characterization. Cell Rep. Phys. Sci..

[16] Das R.S., Agrawal Y.K. (2011). Raman spectroscopy: Recent advancements, techniques, and applications. Vibrat. Spectrosc..

[17] Geraldes C.F. (2020). Introduction to infrared and Raman-based biomedical molecular imaging and comparison with other modalities. Molecules.

[18] Pilot R., Signorini R., Durante C., Orian L., Bhamidipati M., Fabris L. (2019). A review on surface-enhanced Raman scattering. Biosensors.

[19] Moon J., Li M., Ramirez-Cuesta A.J., Wu Z. (2023). Raman Spectroscopy. Springer Handbook of Advanced Catalyst Characterization.

[20] Sun D.-W. (2008). Modern Techniques for Food Authentication.

[21] Jawhari T. (2012). Raman Spectroscopy as A Powerful Analytical Tool: Probing the Structure of Matter. Capítol Del Llibre: Handbook of Instrumental Techniques for Materials Chemical and Biosciences Research.

[22] Simone E., Saleemi A.N., Nagy Z.K. (2014). Application of quantitative Raman spectroscopy for the monitoring of polymorphic transformation in crystallization processes using a good calibration practice procedure. Chem. Eng. Res. Des..

[23] Pirutin S.K., Jia S., Yusipovich A.I., Shank M.A., Parshina E.Y., Rubin A.B. (2023). Vibrational spectroscopy as a tool for bioanalytical and biomonitoring studies. Int. J. Mol. Sci..

[24] Qin J., Kim M.S., Chao K., Cho B.K. (2017). Raman chemical imaging technology for food and agricultural applications. J. Biosyst. Eng..

[25] Cialla-May D., Krafft C., Rösch P., Deckert-Gaudig T., Frosch T., Jahn I.J., Popp J. (2021). Raman spectroscopy and imaging in bioanalytics. Anal. Chem..

[26] Yaseen T., Sun D.W., Cheng J.H. (2017). Raman imaging for food quality and safety evaluation: Fundamentals and applications. Trends Food Sci. Technol..

[27] Avdanina D.A., Zhgun A.A. (2024). Rainbow code of biodeterioration to cultural heritage objects. Herit. Sci..

[28] Prado Araujo F., Hulsbosch N., Muchez P. (2021). High spatial resolution Raman mapping of complex mineral assemblages: Application on phosphate mineral sequences in pegmatites. J. Raman Spectrosc..

[29] Gordon K.C., McGoverin C.M. (2011). Raman mapping of pharmaceuticals. Int. J. Pharm..

[30] Harth A. (2024). The study of pigments in cultural heritage: A review using machine learning. Heritage.

[31] Pezzotti G. (2021). Raman spectroscopy in cell biology and microbiology. J. Raman Spectrosc..

[32] Esmonde-White F.W., Morris M.D. (2009). Raman imaging and Raman mapping. Emerging Raman Applications and Techniques in Biomedical and Pharmaceutical Fields.

[33] He X., Wang J., Gao C., Liu Y., Li Z., Li N., Xia J. (2021). Differentiation of white architectural paints by microscopic laser Raman spectroscopy and chemometrics. Spectrochim. Acta Part A Mol. Biomol. Spectrosc..

[34] Cialla D., März A., Böhme R., Theil F., Weber K., Schmitt M., Popp J. (2012). Surface-enhanced Raman spectroscopy (SERS): Progress and trends. Anal. Bioanal. Chem..

[35] Petersen M., Yu Z., Lu X. (2021). Application of Raman spectroscopic methods in food safety: A review. Biosensors.

[36] Langer J., Jimenez de Aberasturi D., Aizpurua J., Alvarez-Puebla R.A., Auguié B., Baumberg J.J., Liz-Marzán L.M. (2019). Present and future of surface-enhanced Raman scattering. ACS Nano.

[37] Casadio F., Daher C., Bellot-Gurlet L. (2016). Raman Spectroscopy of cultural heritage Materials: Overview of Applications and New Frontiers in Instrumentation, Sampling Modalities, and Data Processing. Top. Curr. Chem..

[38] Pozzi F., Leona M. (2016). Surface-enhanced Raman spectroscopy in art and archaeology. J. Raman Spectrosc..

[39] Cesaratto A., Leona M., Pozzi F. (2019). Recent advances on the analysis of polychrome works of art: SERS of synthetic colorants and their mixtures with natural dyes. Front. Chem..

[40] McNay G., Eustace D., Smith W.E., Faulds K., Graham D. (2011). Surface-enhanced Raman scattering (SERS) and surface-enhanced resonance Raman scattering (SERRS): A review of applications. Appl. Spectrosc..

[41] Moldovan R., Vereshchagina E., Milenko K., Iacob B.C., Bodoki A.E., Falamas A., Bodoki E. (2022). Review on combining surface-enhanced Raman spectroscopy and electrochemistry for analytical applications. Anal. Chim. Acta.

[42] Littleford R.E., Cunningham D., Matousek P., Towrie M., Parker A.W., Khan I., Smith W.E. (2005). Surface-enhanced resonance Raman scattering using pulsed and continuous-wave laser excitation. J. Raman Spectrosc..

[43] Holub D., Pořízka P., Kizovský M., Prochazka D., Samek O., Kaiser J. (2022). The potential of combining laser-induced breakdown spectroscopy and Raman spectroscopy data for the analysis of wood samples. Spectrochim. Acta B.

[44] Zhu X., Xu T., Lin Q., Duan Y. (2014). Technical development of Raman spectroscopy: From instrumental to advanced combined technologies. Appl. Spectrosc. Rev..

[45] Han D., Kim D., Choi S., Yoh J.J. (2017). A novel classification of polymorphs using combined LIBS and Raman spectroscopy. Curr. Opt. Photonics.

[46] Edwards H.G., Vandenabeele P., Colomban P. (2023). Raman Spectroscopy in Cultural Heritage Preservation.

[47] Coccato A., Caggiani M.C. (2024). An overview of Principal Components Analysis approaches in Raman studies of cultural heritage materials. J. Raman Spectrosc..

[48] Manzano E., García-Atero J., Dominguez-Vidal A., Ayora-Cañada M.J., Capitán-Vallvey L.F., Navas N. (2012). Discrimination of aged mixtures of lipidic paint binders by Raman spectroscopy and chemometrics. J. Raman Spectrosc..

[49] Peris-Díaz M.D., Łydżba-Kopczyńska B., Sentandreu E. (2018). Raman Spectroscopy Coupled to Chemometrics to Discriminate Provenance and Geological Age of Amber. J. Raman Spectrosc..

[50] Pereira F.J., López R., Ferrer N., Prieto A.C., Nogal R.A., Nodar A., Aller A.J. (2021). A Comparative Appraisal of Raman Band Ratioing and Chemometric Analysis for Classification of Ancient Papyri. J. Cult. Herit..

[51] Edwards H.G.M., Munshi T. (2005). Diagnostic Raman Spectroscopy for the Forensic Detection of Biomaterials and the Preservation of Cultural Heritage. Anal. Bioanal. Chem..

[52] Aramendia J., Gómez-Nubla L., Fdez-Ortiz de Vallejuelo S., Castro K., Arana G., Madariaga J.M. (2019). The Combination of Raman Imaging and LIBS for Quantification of Original and Degradation Materials in Cultural Heritage. J. Raman Spectrosc..

[53] Festa G., Scatigno C., Armetta F., Saladino M.L., Ciaramitaro V., Nardo V.M., Ponterio R.C. (2022). Chemometric Tools to Point out Benchmarks and Chromophores in Pigments through Spectroscopic Data Analyses. Molecules.

[54] Navas N., Romero-Pastor J., Manzano E., Cardell C. (2010). Raman Spectroscopic Discrimination of Pigments and Tempera Paint Model Samples by Principal Component Analysis on First-Derivative Spectra. J. Raman Spectrosc..

[55] Marengo E., Robotti E., Liparota M.C., Gennaro M.C. (2004). Monitoring of Pigmented and Wooden Surfaces in Accelerated Ageing Processes by FT-Raman Spectroscopy and Multivariate Control Charts. Talanta.

[56] Pallipurath A., Vofély R.V., Skelton J., Ricciardi P., Bucklow S., Elliott S. (2014). Estimating the Concentrations of Pigments and Binders in Lead-Based Paints Using FT-Raman Spectroscopy and Principal Component Analysis. J. Raman Spectrosc..

[57] Offroy M., Marchetti M., Kauffmann T.H., Bourson P., Duponchel L., Savarese L., Mechling J.M. (2024). Using Clustering as Pre-Processing in the Framework of Signal Unmixing for Exhaustive Exploration of Archaeological Artefacts in Raman Imaging. Talanta.

[58] Scatigno C., Prieto-Taboada N., García-Florentino C., Fdez-Ortiz de Vallejuelo S., Maguregui M., Madariaga J.M. (2018). Combination of in Situ Spectroscopy and Chemometric Techniques to Discriminate Different Types of Roman Bricks and the Influence of Microclimate Environment. Environ. Sci. Pollut. Res..

[59] Piantanida G., Menart E., Bicchieri M., Strlič M. (2013). Classification of Iron-Based Inks by Means of Micro-Raman Spectroscopy and Multivariate Data Analysis. J. Raman Spectrosc..

[60] Romero-Pastor J., Cardell C., Yebra-Rodríguez Á., Rodríguez-Navarro A.B. (2013). Validating Chemical and Structural Changes in Painting Materials by Principal Component Analysis of Spectroscopic Data Using Internal Mineral Standards. J. Cult. Herit..

[61] Quintero Balbas D., Lanterna G., Cirrincione C., Ricci M., Becucci M., Fontana R., Striova J. (2022). Noninvasive Identification of Turmeric and Saffron Dyes in Proteinaceous Textile Fibres Using Raman Spectroscopy and Multivariate Analysis. J. Raman Spectrosc..

[62] Wiggins M.B., Liu M., Liu C., Booksh K.S. (2021). Polymorph Identification in Green Chinese Architectural Paints Using Raman Imaging and Multivariate Curve Resolution. J. Chemom..

[63] Bianchi F., Riboni N., Trolla V., Furlan G., Avantaggiato G., Iacobellis G., Careri M. (2016). Differentiation of Aged Fibers by Raman Spectroscopy and Multivariate Data Analysis. Talanta.

[64] Zou Y., Zhang A., Wang X., Yang L., Ding M. (2024). Comparison of Feature Selection and Data Fusion of Fourier Transform Infrared and Raman Spectroscopy for Identifying Watercolor Ink. J. Forensic Sci..

[65] Marengo E., Robotti E., Liparota M.C., Gennaro M.C. (2003). A Method for Monitoring the Surface Conservation of Wooden Objects by Raman Spectroscopy and Multivariate Control Charts. Anal. Chem..

[66] Quintero Balbas D., Lanterna G., Cirrincione C., Fontana R., Striova J. (2022). Non-Invasive Identification of Textile Fibres Using near-Infrared Fibre Optics Reflectance Spectroscopy and Multivariate Classification Techniques. Eur. Phys. J. Plus.

[67] Romero-Pastor J., Cardell C., Manzano E., Yebra-Rodríguez Á., Navas N. (2011). Assessment of Raman Microscopy Coupled with Principal Component Analysis to Examine Egg Yolk-Pigment Interaction Based on the Protein C-H Stretching Region (3100–2800 cm^−1^). J. Raman Spectrosc..

[68] Godinho R.B., Santos M.C., Poppi R.J. (2016). Determination of Fragrance Content in Perfume by Raman Spectroscopy and Multivariate Calibration. Spectrochim. Acta A Mol. Biomol. Spectrosc..

[69] Veneranda M., Irazola M., Díez M., Iturregui A., Aramendia J., Castro K., Madariaga J.M. (2014). Raman Spectroscopic Study of the Degradation of a Middle Age Mural Painting: The Role of Agricultural Activities. J. Raman Spectrosc..

[70] Carlesi S., Picollo M., Ricci M., Becucci M. (2018). The Ageing of Model Pigment/Linseed Oil Systems Studied by Means of Vibrational Spectroscopies and Chemometrics. Vib. Spectrosc..

[71] Henrik-Klemens Å., Abrahamsson K., Björdal C., Walsh A. (2020). An in Situ Raman Spectroscopic Method for Quantification of Polyethylene Glycol (PEG) in Waterlogged Archaeological Wood. Holzforschung.

[72] Pitarch À., Ruiz J.F., Fdez-Ortiz De Vallejuelo S., Hernanz A., Maguregui M., Madariaga J.M. (2014). In Situ Characterization by Raman and X-Ray Fluorescence Spectroscopy of Post-Paleolithic Blackish Pictographs Exposed to the Open Air in Los Chaparros Shelter (Albalate Del Arzobispo, Teruel, Spain). Anal. Methods.

[73] Coccato A., Jehlicka J., Moens L., Vandenabeele P. (2015). Raman Spectroscopy for the Investigation of Carbon-Based Black Pigments. J. Raman Spectrosc..

[74] González Vidal J.J. (2012). Chemometrics in Raman Spectroscopy Applied to Art Works Analysis: Automatic Identification of Artistic Pigments.

